# Orthopoxvirus-specific antibodies wane to undetectable levels 1 year after MVA-BN vaccination of at-risk individuals, the Netherlands, 2022 to 2023

**DOI:** 10.2807/1560-7917.ES.2024.29.38.2400575

**Published:** 2024-09-19

**Authors:** Leanne PM van Leeuwen, Marc C Shamier, Babs E Verstrepen, Hannelore M Götz, Katharina S Schmitz, Najlae Akhiyate, Koen Wijnans, Susanne Bogers, Martin E van Royen, Eric CM van Gorp, Marion PG Koopmans, Rory D de Vries, Corine H GeurtsvanKessel, Luca M Zaeck

**Affiliations:** 1 Department of Viroscience, Erasmus University Medical Center, Rotterdam, the Netherlands; 2 Travel Clinic, Erasmus University Medical Center Rotterdam, the Netherlands; 3 Department of Infectious Disease Control, Municipal Public Health Service Rotterdam–Rijnmond (GGD Rotterdam-Rijnmond), Rotterdam, the Netherlands; 4 Department of Pathology, Erasmus University Medical Center, Rotterdam, the Netherlands; * These authors contributed equally to this work and share first authorship.; ** These authors contributed equally to this work and share senior authorship.

**Keywords:** mpox, monkeypox virus, orthopoxvirus, MVA, vaccination, immune response, antibodies, longevity

## Abstract

In response to the mpox outbreak in 2022 and 2023, widespread vaccination with modified vaccinia Ankara-Bavarian Nordic (MVA-BN, also known as JYNNEOS or Imvanex) was initiated. Here, we demonstrate that orthopoxvirus-specific binding and MVA-neutralising antibodies waned to undetectable levels 1 year post vaccination in at-risk individuals who received two doses of MVA-BN administered subcutaneously with an interval of 4 weeks, without prior smallpox or mpox vaccination. Continuous surveillance is essential to understand the impact of declining antibody levels.

Following an upsurge of mpox cases in parts of Africa associated with the emergence of clade Ib monkeypox virus (MPXV), the World Health Organization (WHO) re-classified mpox a public health emergency of international concern in August 2024 [1-5]. During the global outbreak of clade IIb MPXV in 2022 and 2023, with the vast majority of MPXV infections diagnosed in sexually active gay, bisexual, or other men who have sex with men (GBMSM), many high-income countries initiated vaccination campaigns using the third-generation smallpox vaccine modified vaccinia Ankara-Bavarian Nordic (MVA-BN, also known as JYNNEOS or Imvanex (Bavarian Nordic, Denmark) [6]). Little is known about the durability of antibody responses, and the correlation between waning immunity and long-term effectiveness of MVA-BN vaccination. In this report, we investigated the durability of orthopoxvirus-specific antibody responses and report the longitudinal antibody dynamics up to 418 days after MVA-BN vaccination in two risk groups, namely GBMSM and laboratory workers.

## Description of cohorts

We used sera from two biobank protocols (for methodological details on the study see the Supplement). Baseline characteristics of participants in both studies are documented in Supplementary Table S1.

In the COVA study, sera were collected from 99 high-risk GBMSM and 19 laboratory workers in the Netherlands in 2022 and 2023. Samples were obtained before and after MVA-BN vaccination (V0 = baseline; V1 = 14 days after first dose; V2 = 28 days after first dose; V3 = 28 days after second dose; V4 = 1 year follow-up); the timing of the study visits can be viewed in Supplementary Table S2A. Participants were vaccinated according to the prescribed vaccination regimen: two subcutaneous doses with an interval of 4 weeks for individuals who had not received historic smallpox vaccination. This included participants born in or after 1974 (cessation of smallpox vaccination in the Netherlands), and those born before 1974 with unclear vaccination history (n = 82). Other individuals (n = 17) who declared having received historic smallpox vaccination received one MVA-BN dose. Laboratory workers (n = 19) received two doses of MVA-BN regardless of vaccination history.

In the RestPlus study, residual pseudonymised sera obtained in September 2022 from GBMSM attending the Centre for Sexual Health (CSH) in Rotterdam were screened for the presence of vaccinia virus (VACV)-specific binding antibodies [7]. In case of a positive result, we tested all sera collected from that individual between January 2022 and September 2023, and linked the data to available vaccination and/or infection data. Individuals without registration of an administered MVA-BN vaccine or without a positive MPXV PCR were excluded from further analysis. We included longitudinal serum samples from 116 GBMSM who received MVA-BN vaccination (n = 110) and/or had a positive MPXV PCR (n = 6).

## Durability of orthopoxvirus-specific antibody responses induced by MVA-BN vaccination in at-risk individuals

To determine the longevity of antibody responses, we measured VACV-specific binding antibody levels in a total of 234 participants. In addition, we measured neutralising antibodies (nAbs) against recombinant MVA expressing green fluorescent protein (rMVA-GFP) in sera obtained from the COVA cohort. Assays were performed as previously described [8] and are outlined in the Supplement.

**Figure 1:**
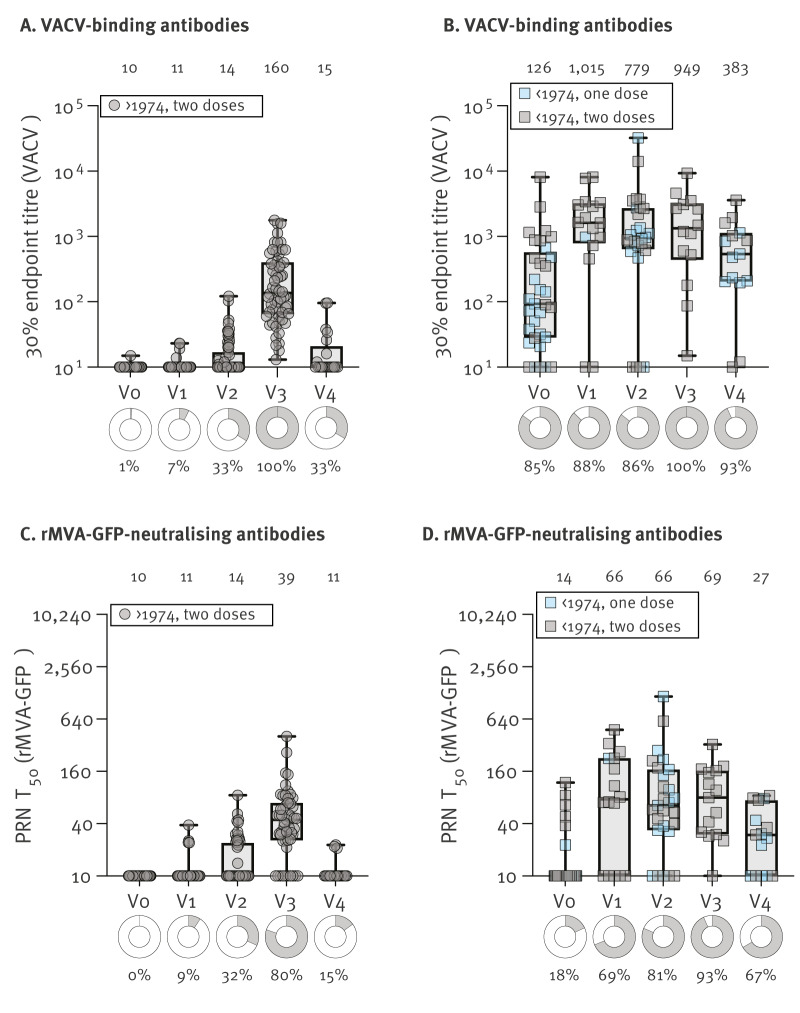
Antibody longevity after MVA-BN vaccination in gay, bisexual, or other men who have sex with men and in laboratory workers, the Netherlands, 2022–2023 (n = 118)

No VACV-specific binding antibodies were detected at baseline in COVA participants born in or after 1974 (Figure 1A). Binding antibody levels gradually increased over time with detectable antibodies in all sera (58/58) collected 4 weeks after the second dose (geometric mean titre (GMT): 160; 95% CI: 116–220). However, only seven of 21 participants had detectable VACV-specific binding antibody levels above the lower limit of detection (LLoD) at 1 year after the second dose (GMT: 15; 95% CI: 11–21). We did not observe a difference in binding antibody kinetics post vaccination between GBMSM and laboratory workers; graphs showing antibody longevity are appended in Supplementary Figure S2.

rMVA-GFP: recombinant MVA expressing green fluorescent protein; MVA-BN: modified vaccinia Ankara-Bavarian Nordic; nABs: neutralising antibodies; PRNT: plaque reduction neutralizing titre; VACV: vaccinia virus

VACV-specific binding or rMVA-GFP nAbs were measured in 118 sera obtained from the COVA cohort. Individuals born from 1974 onwards are presumed to be immunologically naive, those born before 1974 are presumed to have received historic smallpox vaccination.

Panel A: VACV-specific binding antibody levels in immunologically naive participants after receiving two doses of MVA-BN. Panel B: VACV-specific binding antibody levels in participants with pre-existing immunity after receiving either one dose or two doses of MVA-BN. One participant born before 1974 deviated from the study protocol and had a V1 instead of a V2 visit. Panels C and D: rMVA-GFP nAbs after receiving one or two doses of MVA-BN.

Data are shown in box-and-whisker plots, with the horizontal lines indicating the median, the bounds of the boxes indicating the interquartile range, and the whiskers indicating the range. Bold numbers above the plots represent the geometric mean titre per time point. The percentage of positive samples are shown as doughnut graphs and numerical values below each time point. V0 = baseline; V1 = 14 days after first dose; V2 = 28 days after first dose; V3 = 28 days after second dose; V4 = 1 year follow-up.

In individuals born before 1974, VACV-specific binding antibodies were detected at baseline before MVA-BN vaccination in 28 of 33 participants (Figure 1B); detailed endpoint titres are appended in Supplementary Table S2B. Binding antibody levels were boosted by the first dose of MVA-BN (GMT: 897; 95% CI: 347–2,324 for those receiving one dose, and GMT: 675; 95% CI: 166–2,742 for those receiving two doses) at 28 days after first vaccination. A second dose of MVA-BN did not result in an additional increase of binding antibody levels (GMT: 949; 95% CI: 347–2,596 at 28 days after second vaccination; Figure 1B). Binding antibody levels in MVA-BN-vaccinated participants with prior immunity remained stable at the 1-year follow-up. Two participants consistently had low or undetectable antibody levels for unknown reasons.

To assess the durability of antibody functionality, we measured rMVA-GFP nAbs in participants of the COVA study. In 41 of 51 participants born in or after 1974, we detected rMVA-GFP nAbs 28 days after the second MVA-BN vaccination (GMT: 39; 95% CI: 31–51), compared with only three of 20 participants 1 year post vaccination (GMT: 11; 95% CI: 9.8–13) (Figure 1C). In participants born before 1974, rMVA-GFP nAbs were boosted by the first dose of MVA-BN, without an additional increase after the second dose, comparable to binding antibodies (Figure 1D). One year post vaccination, rMVA-GFP nAb levels waned, with five of 15 individuals having rMVA-GFP nAb levels below the LLoD.

**Figure 2:**
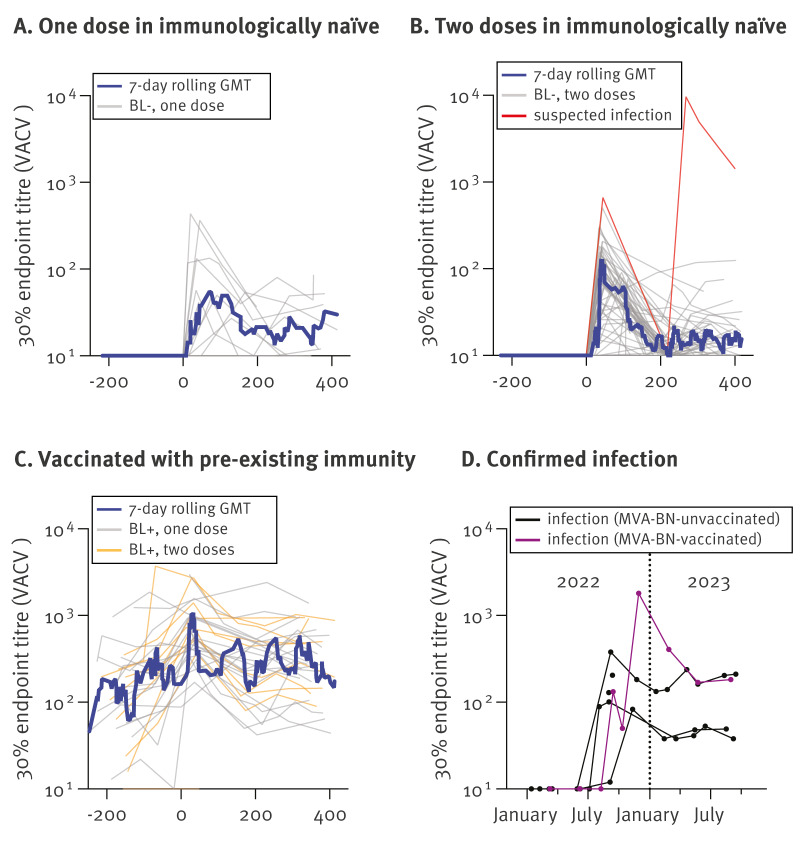
Antibody longevity after MVA-BN vaccination in longitudinal serum samples obtained from gay, bisexual, or other men who have sex with men visiting the Centre for Sexual Health in Rotterdam, the Netherlands, January 2022–September 2023 (n = 116 individuals)

BL: baseline; GMT: geometric mean titre; MVA-BN: modified vaccinia Ankara-Bavarian Nordic; VACV: vaccinia virus.

We additionally studied antibody kinetics up to 418 days post vaccination in longitudinal serum samples obtained from 116 GBMSM clients of the Rotterdam CSH who had received MVA-BN vaccination (n = 110) or had a positive MPXV PCR (n = 6). The vaccinated participants were grouped based on the absence or presence of VACV-specific binding antibodies on the day of MVA-BN vaccination into a baseline-negative (Figure 2A-B) and a baseline-positive (Figure 2C) group. Independent of the status at baseline, all groups showed an increase in binding antibody levels post vaccination, which peaked within 30–90 days. Subsequently, binding antibody levels began to decline across all groups, returning close to their respective baseline levels 1 year post vaccination. For baseline-negative study participants, among those who were followed-up for a year or longer, two-thirds (22/34) had low (< 20) to no detectable antibodies at the end of the study, whereas baseline-positive individuals returned to their original baseline levels.

VACV-specific binding antibody levels were measured in sera obtained from 116 GBMSM clients of the Centre for Sexual Health in Rotterdam, the Netherlands, as part of the RestPlus cohort.

Panels A and B: VACV-specific binding antibody levels in RestPlus participants without detectable antibodies at baseline (BL-). Panel C: VACV-specific binding antibody levels in RestPlus participants with detectable antibodies at baseline (BL+) and either one or two doses of MVA-BN. Panel D: VACV-specific binding antibody levels in RestPlus participants with a PCR-confirmed MPXV infection. All individuals were MVA-BN-unvaccinated except for one.

## Antibody kinetics of monkeypox virus infections among gay, bisexual, or other men who have sex with men

In the cohort of the Rotterdam CSH, there were six participants with a PCR-confirmed MPXV infection during the study period, all born after 1974 (Figure 2D). All infections occurred during the global clade IIb outbreak in 2022 and 2023. For two of these individuals, only a single serum sample was available. One participant was diagnosed with an MPXV infection in October 2022, following the completion of a two-dose MVA-BN vaccination regimen in early September 2022 (Figure 2D, purple line). Comparison of this individual’s binding antibody levels shortly before and 1 month after infection revealed an increase from 50 to 1,807. Based on the binding antibody kinetics and magnitude, we suspect an additional undetected infection among one of the participants after two doses of MVA-BN (Figure 2C, red line).

## Discussion

Here, we showed that MVA-BN vaccination induced binding and rMVA-GFP-neutralising antibodies in previously unvaccinated at-risk study participants (born in 1974 or later), and that vaccination boosts binding antibody levels in historically vaccinated at-risk individuals (born before 1974). Binding and neutralising antibody levels in non-primed at-risk individuals declined rapidly at 1-year follow-up and became undetectable in a considerable proportion of cases.

Measuring the immunogenicity of MVA-BN is crucial for understanding the impact of immunisation strategies and supporting vaccine effectiveness (VE) studies. We have previously shown that a two-dose MVA-BN vaccination regimen is immunogenic in individuals without pre-existing immunity [8]; vaccination resulted in the production of VACV-specific binding antibodies, rMVA-GFP nAbs, and MPXV nAbs 1 month after the second dose. However, the levels of MPXV nAbs were low compared with those after historic smallpox vaccination or after MPXV infection [8]. In the initial months after the start of MVA-BN vaccination, VE studies yielded an estimated aggregate VE of 82% after two doses [9,10]. However, while antibodies induced by historic smallpox vaccination using first- or second-generation vaccines can be detected for decades [11], we find rapid waning of antibody in vaccine recipients without pre-existing immunity, which aligns with prior studies into the longevity of orthopoxvirus-specific antibodies after MVA-BN vaccination [12-16]. We expand on those by providing longitudinal immunological follow-up of over 1 year in those directly at risk of MPXV infection. While the clinical significance of low to absent antibody levels 1 year after vaccination remains unclear due to the absence of a clearly defined correlate of protection, waning antibody levels raise the important question whether these individuals are still protected and if this could possibly facilitate mpox resurgence. A recent report suggested that the level of circulating titres might indeed not be the only marker of protection and that other parts of the immune system, including the role of cell-mediated immunity and the robustness of memory responses, might be more important determinants of disease outcome [17].

Combined with the continuing burden of MPXV, this highlights the importance of further investigating the long-term efficacy of MVA-BN. Ongoing circulation of clade I MPXV on the African continent, including the emergence of clade Ib [4], necessitates future studies elaborating on the cross-reactivity of vaccine-induced antibody responses against clade Ia/b MPXV [5]. In addition, administration of a booster dose 1 year after the initial dose could be considered to improve vaccine immunogenicity [8,15].

## Conclusion

In this study, we demonstrate a rapid decline in binding and neutralising antibodies 1 year after MVA-BN vaccination in non-primed at-risk individuals. Taken together, our data contribute to the understanding of waning humoral immune responses following MVA-BN vaccination and support decision-making on vaccination strategies.

## Supplementary Data

875000 Supplement
